# IEEE 802.11ah: A Technology to Face the IoT Challenge

**DOI:** 10.3390/s16111960

**Published:** 2016-11-22

**Authors:** Victor Baños-Gonzalez, M. Shahwaiz Afaqui, Elena Lopez-Aguilera, Eduard Garcia-Villegas

**Affiliations:** 1 Department of Network Engineering, Universitat Politècnica de Catalunya BarcelonaTech, C/Esteve Terradas, 7, Castelldefels 08860, Spain; elopez@entel.upc.edu (E.L.-A.); eduardg@entel.upc.edu (E.G.-V.); 2 Department of Electronics Engineering, Maynooth University, Maynooth, Kildare W23 F2H6, Ireland; shahwaiz.afaqui@nuim.ie

**Keywords:** 802.11ah, IoT, applications

## Abstract

Since the conception of the Internet of things (IoT), a large number of promising applications and technologies have been developed, which will change different aspects in our daily life. This paper explores the key characteristics of the forthcoming IEEE 802.11ah specification. This future IEEE 802.11 standard aims to amend the IEEE 802.11 legacy specification to support IoT requirements. We present a thorough evaluation of the foregoing amendment in comparison to the most notable IEEE 802.11 standards. In addition, we expose the capabilities of future IEEE 802.11ah in supporting different IoT applications. Also, we provide a brief overview of the technology contenders that are competing to cover the IoT communications framework. Numerical results are presented showing how the future IEEE 802.11ah specification offers the features required by IoT communications, thus putting forward IEEE 802.11ah as a technology to cater the needs of the Internet of Things paradigm.

## 1. Introduction

**Figure 1:**
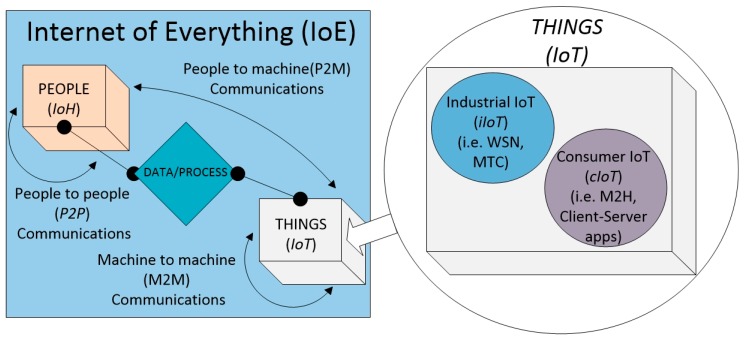
Internet of Everything concept.

In recent years, we have witnessed an exponential growth in the evolution and development of different communication technologies addressed to support the IoT. New applications require innovative connectivity solutions and new ways of sharing data among different devices and networks, thus creating a new concept of Internet. In the related literature, a collection of new terms have been coined in an attempt to clarify the new scenario of connected applications, the Internet of Everything (IoE) (cf. Figure 1) appears as a concept that contains both the IoT and the Internet of Humans (IoH), including the capability to share data between each other (IoT and IoH) or among themselves using machine to machine (M2M) or machine to human (M2H) communications. Following a similar approach, we could shape the IoT definition to include two different concepts: industrial IoT (iIoT) and consumer IoT (cIoT), exhibiting a new scenario that will dominate the world’s communications in the near future, at least in terms of number of participating devices.

The upcoming IoT applications are enablers of innovative concepts such as smart cities, smart/e-health, smart metering and smart things. Each of these applications has particular requirements, i.e., different data rates, low power consumption, low cost of implementation, large number of supported devices and the capacity to cover different distance ranges. As signified in [1], it is estimated that the number of devices connected to the IoT will reach 50 billion by 2020. This massive implementation of the IoT paradigm will bring changes to many aspects of our lives. The debate on which technology should lead this revolution has not been settled yet. Over the years there were multiple contenders, while Wi-Fi seemed to be observing from the bench.

The legacy IEEE 802.11 standard was originally developed for indoor home and office scenarios with recognized worldwide success. Nowadays, IEEE 802.11 can be considered as a ubiquitous technology found in a wide range of consumer electronic devices and used in heterogeneous scenarios. However, up till now, IEEE 802.11 has not shown a significant presence in the IoT market, without any specification focused on IoT and its singularities. Taking into account the near future scenario for IoT communications, IEEE 802.11 Working Group (WG) aims to bridge the gap by introducing the new amendment called IEEE 802.11ah [2]. Based on the IEEE 802.11ah, the Wi-Fi Alliance has recently introduced the Wi-Fi HaLow program and expects to launch the certification process in 2018. Therefore, IEEE 802.11ah is the first approximation of IEEE 802.11 WG that can enable IoT specific features within thousands of stations operating at Sub 1GHz Industrial, Scientific and Medical (ISM) frequency band [3,4,5]. The amendment has been designed based on the following uses cases:

- *Smart sensors and meters.* The goal of the new amendment is to enable IEEE 802.11 technology to cover IoT applications for indoor and outdoor spaces in urban, suburban and rural environments.
- *Backhaul aggregation.* This is a scenario in which IEEE 802.11ah routers/gateways would gather data from leaf devices (i.e., sensors) and forward information to servers, utilizing IEEE 802.11ah links. This use case is attractive for long range communications.
- *Extended range hotspot and cellular offloading.* Both high throughput and long transmission range make sub-1 GHz communications very attractive for extending hotspot range and for traffic offloading in mobile networks.

Most recently, the IEEE 802.11 WG has triggered other future specifications to include the IoT use case. As of July 2015, the creation of a new Topic Interest Group (TIG) on Long-Range Low-Power (LRLP) operation for IoT was initiated [6], which aimed to bring some of the new IEEE 802.11ah features to the 2.4 GHz band while keeping compatibility with mainstream IEEE 802.11 devices on that band. In May 2016 the TIG agreed to focus on the issue of low power (leaving aside the long range feature), creating a Study Group (SG), the LP-WUR (low-power wake-up receiver) SG. Therefore, the LRLP TIG has been dissolved.

Besides, IEEE 802.11 initiated the task group TGax that aims at investigating as well as delivering next generation WLAN technologies and at characterizing PHY along with MAC modifications/amendments to improve performance and, thus, energy efficiency in transmission mechanisms. New proposals are being explored by TGax to accommodate the IoT use case [7], and thus, to adopt some of the LRLP propositions. The forthcoming IEEE 802.11ax amendment is expected by 2019.

It is well known that IEEE 802.11 specifies the mechanisms corresponding to MAC and PHY layers. On the other hand, the Internet Engineering Task Force (IETF) is in charge of the Internet standards development, being responsible of the first reference protocol stack for the IoT after a decade of work, which includes the adaptation layer 6LoWPAN to support IPv6 over IEEE 802.15.4 networks. New adaptation layers are being proposed in the IETF 6Lo Working Group, such as the one addressed to get an efficient transport of IPv6 packets over IEEE 802.11ah [8].

IEEE 802.11ah is not the only technology trying to cover the requirements of IoT communications. IEEE 802.11 will have to compete with other technologies that are already established in the IoT arena, such as ZigBee/IEEE 802.15.4e, BLE (Bluetooth Low Energy) and different Low Power Wide Area Network (LPWAN) proprietary technologies. In Table 1, we briefly summarize the most notable characteristics of those technologies.

Each technology presented in Table 1 has particular features that are attractive for different IoT scenarios. ZigBee/IEEE 802.15.4e has been used in most of the Wireless Sensor Networks (WSN) due to its low implementation cost, the large number of supported devices, the offered data rates (i.e., 20 to 250 kbps) and the low power consumption, which makes it attractive for some IoT short-range low-rate applications. Similarly, BLE (which is an amendment of Bluetooth 4.0) is focused on low energy consumption and short-range low-rate communication. At present, Bluetooth Special Interest Group (SIG) is developing next Bluetooth 5 that promises enhancements in data rates and coverage ranges. 3rd Generation Partnership Project (3GPP) through Machine Type Communications (MTC) technology is also making an effort to standardize M2M (Machine to Machine) communications offering features such as Quality of Service (QoS), mobility and roaming support based on cellular technologies. In addition to the higher frequency bands used in 3GPP MTC, the refarming of licensed Global System for Mobile Communications (GSM) spectrum brings the possibility to use sub 1 GHz frequencies. 3GPP MTC, which is mentioned in release 12 and 13 and will be further developed in future releases, presents the largest coverage feature and the highest number of supported devices in comparison to the other aforementioned technologies, but operates in licensed spectrum.

3GPP has also introduced Narrowband Internet of Things (NB-IoT) that allows operators to use a minimal portion of the available spectrum (Long-Term Evolution, LTE, or GSM networks) to target ultra-low-end IoT applications. However, NB-IoT is expected to suffer from not being full backward compatible with existing 3GPP devices. It is anticipated that this specification will be completed in 2016 [9]. In addition, in the past few years, LPWAN solutions have appeared in competition to conquer the IoT market. Probably, the most outstanding solutions nowadays are LoRa and SigFox which present long coverage ranges (less than 3GPP MTC) and increased number of supported devices [10].

In comparison to the foregoing technologies, IEEE 802.11 presents low implementation cost and consists in a widely spread technology deployed in many consumer electronic devices. Shipments of IEEE 802.11 devices reached 12 billion just at the beginning of 2016, and will reach 15 billion by the end of 2016, according to current predictions (information extracted from Wi-Fi Alliance). Current literature on IEEE 802.11ah, however, does not provide enough evidence to support the suitability of this technology in an IoT scenario. In this regard, this paper shows how IEEE 802.11ah can cover the requirements of the most common IoT applications.

## 2. Challenges for IoT Applications and IEEE 802.11ah 

In order to visualize the challenges within IoT communications, we can distinguish the typical requirements such as large number of autonomous devices sending traffic (simultaneously or in deferred times), low power consumption and long sleep time. In this section we provide an overview of the mechanism used by IEEE 802.11ah to tackle these challenges.

### 2.1. Coverage Range

Some of the IoT applications require more than 1 km of coverage for their desired operation. Besides the fact that it is using a lower frequency band (sub-1 GHz) with better propagation characteristics, in IEEE 802.11ah, the extended range requirement is fulfilled by introducing 1 MHz wide transmission and by using a new Modulation and Coding Scheme (MCS) index (MCS10). This scheme is effectively MCS0 (BPSK 1/2) with an addition of 2× repetition. Along with 1 MHz channel bandwidth (CBW), IEEE 802.11ah also supports 2, 4, 8 and 16 MHz (it is expected that early commercial devices support up to 4 MHz). These narrower bandwidths entail a larger symbol duration than legacy IEEE 802.11. With longer symbols (and guard intervals), IEEE 802.11ah transmissions are more robust to inter-symbol interference found in longer links and outdoor scenarios (large delay spread). By supporting Multiple-Input Multiple-Output (MIMO), IEEE 802.11ah benefits from spatial diversity, which improves the received signal quality and, hence, makes longer links possible. The specification also considers multi-hop operation with relays or mesh networking to extend coverage.

### 2.2. Time and Frequency Resources

Many technologies concurrently operate in the overcrowded frequency band of 2.4 GHz (IEEE 802.15.4e, BLE, IEEE 802.11, etc.), where they incur in a lot of interference, which seriously degrades the performance of the network. With the advent of IoT, and the increase in the number of devices implementing these technologies, the fate of this band does not look promising; on the contrary, communications problems, such as the co-channel interference, which is especially harmful in Carrier Sense Multiple Access (CSMA)-like access schemes, will be exacerbated. However, the IEEE 802.11ah amendment is intended to operate below 1 GHz which, besides improved coverage, faces less interference. This characteristic of the IEEE 802.11ah appears particularly attractive for IoT applications, where hundreds or thousands of devices are expected to coexist.

### 2.3. Supporting a Large Number of IoT Devices

IoT networks have the main characteristic of being formed by a large number of autonomous devices (typically ranging from hundreds to few thousands). This is because many of the applications are expected to operate over a large area. However, collisions occur frequently when a large number of devices try to communicate simultaneously. Excessive collisions result in reduced overall throughput in the network and thus, finding appropriate methods to reduce collisions is a challenge for the IoT. The IEEE 802.11ah defines an optional new contention channel access mechanism called Restricted Access Window (RAW). This access method is designed to reduce collisions by improving the channel efficiency by dividing stations into different groups and restricting channel access only to a group at a particular time period.

Legacy IEEE 802.11 supports up to 2007 associated stations per Access Point (AP), due to the limited number of available Association IDentifiers (AID) that can be assigned to each associated station. In order to increase the number of supported stations by AP, IEEE 802.11ah utilizes a novel hierarchical AID structure. The new AID consists of 13 bits and thus the number of supported stations increases to 2^13^ − 1 (8191). AID structure consists of four hierarchical levels (i.e., page, block, sub-block, and station’s index in sub-block). IEEE 802.11ah employs the aforementioned structure to group stations based on similar characteristics (e.g., traffic pattern, location, battery level, etc.).

### 2.4. Low Power Consumption

Considering the fact that many IoT devices are battery driven and are meant to operate for days, weeks, months or years (depending on the application), the low power consumption becomes a crucial aspect to increase the battery life. IoT devices are equipped with embedded Network Interface Card (NIC) and thus have the ability to communicate autonomously within the network they belong to. The wireless NIC represents a large portion of the energy consumed by the device and thus, the definition of an efficient power management for the NIC is of paramount importance. This can be achieved by employing different wake up and doze timers.

In legacy IEEE 802.11, the specified maximum idle period allows any station to maintain its association state for up to 18.64h of inactivity, while IEEE 802.11ah aims to utilize different periods for different applications, up to a year scale.

Many new features introduced by the IEEE 802.11ah are intended to achieve more efficient transmissions, thus allowing energy savings. For example, the reduced overhead due to shorter headers and mechanisms such as the implicit acknowledgement (ACK control frames not required in some cases), the speed frame exchange (method that allows to exchange a bidirectional sequence of frames during a reserved Transmit Opportunity (TXOP)), extend battery life of stations by shortening transmission time, keeping them awake for shorter periods.

## 3. Comparative Analysis of IEEE 802.11ah with Previous IEEE 802.11 Amendments

In this section, we present a comparison between IEEE 802.11ah and different IEEE 802.11 amendments. First, we describe the differences between IEEE 802.11 amendments based on MAC features. Later, we provide performance comparison between IEEE 802.11ah and the previous IEEE 802.11 amendments in terms of throughput versus transmission range characteristics.

### 3.1. Comparison of IEEE 802.11 Amendments Based on MAC Features

IEEE 802.11ah’s physical layer is basically an adaptation of IEEE 802.11ac to the sub-1 GHz band. The physical layer is a 10 times down-clocked version of IEEE 802.11ac (symbol duration from 4 to 40 µs), which keeps the same number of OFDM subcarriers. In consequence, the resulting channel bandwidth is ten times smaller than its IEEE 802.11ac counterpart (i.e., 2, 4, 6, 8 and 16 MHz) and adds a special mode of 1 MHz. As mentioned before, IEEE 802.11ah also defines a more robust MCS (BPSK 1/2 with repetition). The support of up to 4 × 4 MIMO (including multi-user MIMO) can be used to enable spatial diversity and/or spatial multiplexing to increase the capacity of the links and to improve coverage.

The key design feature for the IEEE 802.11 MAC is based on the channel access principle that enforces each station to sense the channel to be idle before initiating transmission, in order to avoid collisions. The MAC operation was designed based on Distributed Coordination Function (DCF) (explained below) protocol that utilizes the aforementioned principle. Despite the robust and adaptive nature of DCF in varying conditions, the initial MAC features were designed for best effort applications and thus did not require complex resource scheduling or management algorithms. However, the massive deployment of IEEE 802.11 networks has resulted in the need to include traffic differentiation and other sophisticated network management schemes. Furthermore, different versions of the IEEE 802.11 standard have been proposed with time, which include additional PHY and MAC features to accommodate the technological advances along with the ability to adapt to ever growing use cases.

Table 2 highlights the key MAC features supported by each amendment. In particular, we highlight the critical MAC additions and changes being made for IEEE 802.11ah, which will allow IEEE 802.11 standard to accommodate the IoT paradigm. The notable features compared in Table 2 are briefly introduced in the following paragraphs.

#### 3.1.1. Backwards Compatibility

Up till IEEE 802.11ac, all the IEEE 802.11 systems have been designed to be backward compatible. However, for IEEE 802.11ah, backward compatibility is not considered due to the use of a completely different frequency band.

#### 3.1.2. Distributed Channel Access (DCF)

It is the basic random access MAC protocol of IEEE 802.11 standard that includes CSMA with Collision Avoidance (CSMA/CA), a sort of listen before talk mechanism. Furthermore, it encompasses binary exponential back-off rules to manage the retransmission of collided frames. It works as follows. Before initiating a transmission, a station senses the channel to determine whether it is busy. If the medium is sensed idle during a period of time called the Distributed Inter-frame Space (*DIFS*), the station is allowed to transmit. If the medium is sensed busy, the transmission is delayed until the channel is idle again. In this case, a slotted binary exponential back-off interval is uniformly chosen in [0, *CW*-1], where *CW* is the contention window. After each data frame is successfully received, the receiver transmits an acknowledgment frame after a Short Inter-frame Space (*SIFS*) period.

#### 3.1.3. Point Coordinated Function (PCF)

It is an optional MAC protocol that uses polling scheme to determine which station can initiate data transmission. This technique is designed for infrastructure based network only, where different stations can optionally participate in PCF and respond to poll received.

#### 3.1.4. Hybrid Coordination Function (HCF)

HCF, which combines the aspects of both the contention based DCF and controlled channel access based PCF, is a Quality of Service (QoS) aware MAC protocol that includes appropriate service differentiation mechanism. HCF defines two methods of channel access.

- HCF Controlled Channel Access (HCCA)
  It is similar to PCF and uses the same polling mechanism to assign transmission opportunity to QoS enabled stations.
- Enhanced Distributed Channel Access (EDCA)
  EDCA is an extension of the DCF mechanism that tries to implement service differentiation by classifying the traffic into different categories with different priorities. In EDCA mode, a traffic class can make itself a higher prioritized traffic class by statistically reducing its transmission delay by declaring an Access Category (AC) that has higher priority for contending shared channel.

#### 3.1.5. Transmission Opportunity (TXOP)

- For IEEE 802.11-2007:
  TXOP defines a period of time for which a station accessing the channel is allowed to transmit multiple frames without using channel access procedure for all the frames.
- For IEEE 802.11n/ac/ah:
  In these amendments, the TXOP procedure is enhanced, where the reverse mechanism allows the holder of TXOP to allocate the unused TXOP time to its receiver to enhance the channel utilization and perform reverse direction traffic flows. This mechanism is known as Reverse Direction (RD) protocol.
- For IEEE 802.11ah:
  IEEE 802.11ah has introduced bi-directional TXOP (BDT) that can help non-AP station (i.e., sensors etc.) to minimize energy consumption. This technique allows the combination of transmission and reception of frames within a single TXOP, where the reduction in the required frame exchange enables stations to extend their battery life time. In addition, this mechanism assists in efficient use of contention based channel accesses.

#### 3.1.6. Response Indication Deferral (RID)

This method is an extension of Virtual carrier sensing mechanism originally defined in legacy IEEE 802.11 (i.e., Network Allocation Vector (NAV)). The short header defined by IEEE 802.11ah does not include the Duration/ID field that is required by the NAV. Both NAV and RID indicate countdown timers used to show the channel idle time. However, the two schemes differ in the procedure to set the counter (while NAV is set after the complete and correct reception of a frame, RID can be set after the complete header of the frame is received).

#### 3.1.7. Frame Aggregation:

Mechanism to combine multiple data frames into one larger aggregated data frame for transmission.

- For IEEE 802.11n:
  It employs two steps of accumulation to increase the size of the data frame to be transmitted. The first, which is at the top of the MAC, assembles MAC service data units (MSDU) and is called A-MSDU. Another, at the bottom of the MAC, adds MAC Protocol Data Units (MPDUs) and is called A-MPDU.
- For IEEE 802.11ac/11ah:
  Enhanced frame aggregation methods are used. All frames follow the A-MPDU format; the maximum size of A-MPDU is increased for IEEE 802.11ac.

#### 3.1.8. Block Acknowledgement (Block ACK)

This mechanism enables the transmission of a single ACK frame by the station that received series of frames. This fact results in efficient use of airtime as compared to traditional positive ACK sent for every received frame.

- For IEEE 802.11n:
  Block ACK method is modified to support multiple MPDUs in an A-MPDU. The sender only resends the MPDUs that have not been correctly received by the receiver and are not acknowledged by it.
- For IEEE 802.11ah:
  Block ACK response includes the preferred MCS and the bandwidth information. IEEE 802.11ah also introduces the fragment Block ACK procedure. Fragments obtained from the partition of a MSDU can be acknowledged either using immediate acknowledgement by responding with NDP Block ACK frames, or following the normal Block ACK procedure.

#### 3.1.9. Multi-User (MU) Aggregation

This method defined by the IEEE 802.11ac, supports the aggregation of MPDUs from multiple receivers into a single PDU only used for transmission from AP to multiple stations.

#### 3.1.10. Null Data Packet (NDP)

Null frame is a frame meant to contain no data but flag information. They are widely used in IEEE 802.11 WLANs for control purposes such as power management, channel scanning, and association keeping alive.

#### 3.1.11. Group ID

This mechanism enables a receiver to determine whether the data payload is single- or multi-user. More specifically, the Group-ID field is utilized by a receiving node to decide if it is targeted in the followed multi-user (MU) MIMO transmission.

#### 3.1.12. BSS Color

It is an innovative scheme to increase throughput of dense WLAN networks, where each BSS is assigned a specific color (in-terms of bits designated in LSIG field of physical header). A station upon receiving frames from neighboring BSS, can abandon the reception process assuming the channel idle during that transmission and thus increasing the transmission opportunities.

#### 3.1.13. Dynamic Bandwidth Management

IEEE 802.11ac has also introduced dynamic bandwidth management to optimize the use of available bandwidth. This scheme allows the transmitter and receiver to select an interference free channel before initiating transmission.

#### 3.1.14. Subchannel Selective Transmission (SST)

This feature has been introduced by IEEE 802.11ah. It allows stations to rapidly select and switch to different channels between transmissions to counter fading over narrow subchannels.

#### 3.1.15. Traffic Indication Map (TIM)

In legacy IEEE 802.11, the Beacon frame contains this element through which the sleeping power saving stations are informed of the presence of buffered traffic intended for them at the AP. This element is sent in the form of a bitmap, where each bit represents the Association ID (AID) of stations. A bit is set in TIM when corresponding station has buffered data at the AP. The Delivery Traffic Indication Message (DTIM) serves a similar purpose, indicating the presence of buffered multicast frames.

#### 3.1.16. Target Wake Time (TWT)

TWT is a function that permits an AP to define a specific time or set of times for individual stations to access the medium.

#### 3.1.17. Hierarchical AID

IEEE 802.11ah proposed hierarchical network organization where stations are grouped together based on their similarities. Each station is assigned a four level AID structure encompassing page, block, sub-blocks and station fields. As an important outcome, this mechanism helps in supporting increased number of stations.

#### 3.1.18. Dynamic AID Reassignment

This mechanism allows the AP to change the page/group of a station due to a change in its traffic characteristics or for load distribution among the channels.

#### 3.1.19. Restricted Access Window (RAW)

It is a new contention-free channel access mechanism that is designed to reduce collisions by improving the channel efficiency. The AP coordinates the uplink channel access of the stations by defining RAW time intervals in which specific class of devices are given exclusive access of the shared medium.

#### 3.1.20. Group Sectorization

This scheme is developed by IEEE 802.11ah that allows stations to transmit in different sectors (positions) around the AP in a time division multiplexing manner (i.e., after each Beacon, a different sector is given access to the shared medium). The Beacons transmitted by a sectorized BSS carry sector option element and each station is allocated a group ID based on sectorization operation.

#### 3.1.21. Relay Operations 

IEEE 802.11ah has defined a mode of operation to utilize relays within the network to facilitate the exchange of frames between stations and APs. Relays allow stations to utilize higher data rates and TXOP sharing.

#### 3.1.22. Power Saving at AP

IEEE 802.11ah proposes to include AP power saving features in IEEE 802.11ah.

#### 3.1.23. Low Power Mode of Operations

IEEE 802.11ah enables a station to inform the AP about the duration of time it intends to remain in sleep mode. During the sleep mode, the station is not intended to listen to Beacons and then it is able to reduce its power consumption.

### 3.2.Throughput and Range Characterization of IEEE 802.11 Amendments

In order to compare different IEEE 802.11 amendments, we evaluate layer-2 throughput versus coverage range by using different channel bandwidth values, number of Spatial Streams (SS) and MCS. We analyze a scenario defined by a single radio link composed of two stations (transmitter and receiver) where we consider path loss models defined by TGah [11]. The macro deployment model assumes an outdoor scenario with antenna placed at 15 m above rooftop. On the other side, we employ the large indoor open space TGah path loss model with Non-Line-of-Sight (NLoS) conditions, which corresponds to a factory/warehouse type of environment. The macro deployment path loss model follows the next expression:

(1) PL=8+37.6log10(d)

where *d* corresponds to the distance in meters between transmitter and receiver, and radio frequency carrier is 900 MHz. For other frequencies, a correction factor of $21\times \log10\left(\frac{f}{900MHz}\right)$ should be applied.

TGah indoor path loss model is modelled by directly scaling down the frequency operations of TGn path loss model. It consists of the free space loss model (slope of 2) up to a breakpoint distance ($d_{BP}$), and employs a slope of 3.5 after the breakpoint. We consider the large indoor open space scenario with NLoS conditions (with $d_{BP}$ of 5 m). This indoor channel model would correspond to a factory/warehouse type of environment:

(2) $$L\left(d\right)=\{\begin{array}{l} L_{FS}\left(d\right)=20\;\log10\left(\frac{4\pi fc}{c}\right)\;d\leq d_{BP}\\ L_{FS}\left(d_{BP}\right)+35\log10\left(\frac{d}{d_{BP}}\right)\;d>d_{BP} \end{array}$$

where *d* corresponds to the distance in meters between transmitter and receiver, fc is the center carrier frequency in MHz and c the speed of the light in m/s. Note that the TGah indoor channel propagation loss model was recently amended according to [12] as shown in Equation (2).

We consider omnidirectional antennas with 0dB gain and transmitted power of 30 dBm. The MAC aggregation feature is included in our evaluation, and ideal transmission conditions have been considered for comparison purposes.

The throughput expression S in Mbps is as follows, employing DCF MAC access and including the aggregation feature:

(3) $$S=\frac{L_{data}\times 8\times K}{T_{message}}$$

where *K* is the number of aggregated frames (of equal size), *L_data_* corresponds to the payload size and *T_message_* is computed as:

(4) $$T_{message}=DIFS+T_{DATA}+SIFS+T_{BA}+T_{BACKOFF}+2\delta$$

*DIFS* and *SIFS* are given in Table 3, δ is the propagation delay, *T_BA_* corresponds to the duration of an Block ACK frame and *T_DATA_* represents the transmission time of a data frame, which depends mainly on the size of the payload and on the PHY rate. *T_DATA_* and *T_BA_* computation also depends on the IEEE 802.11 amendment used in the transmission. Under ideal channel conditions, we consider that $T_{BACKOFF}$ is *CW_min_*/2 times the slot time (T_Slot_); *CW_min_* corresponds to the minimum *CW* (cf. Table 3). All frame sizes are given in Bytes and frame durations in µs.

*T_DATA_* calculation for IEEE 802.11ah includes three different cases:

1. 1 MHz CBW case with short and long Guard Interval (GI) subcases, following Equations (5) and (6), respectively. Note that with 1 MHz CBW only one PHY preamble/header type applies (cf. Table 3).
2. Short preamble case for 2, 4, 8 and 16 MHz CBW with short and long GI subcases, which also follow Equations (5) and (6), respectively; in this case, a different value for the PHY preamble/header length should be used (cf. Table 3).
3. Long preamble case for 4, 8 and 16MHz CBW with short and long GI subcases, following Equations (7) and (8), respectively:
  (5) $$T_{shortGI}=T_{Preamble\&Header}+40\times \left(N_{LTF}-1\right)+T_{Syml}+T_{Syms}\times \left(N_{sym}-1\right)$$
  (6) $$T_{longGI}=T_{Preamble\&Header}+40\times \left(N_{LTF}-1\right)+T_{Syml}\times N_{sym}$$
  (7) $$T_{shortGI\;long\_preamble}=T_{Preamble\&Header}+40\times N_{LTF}+T_{Syml}+T_{Syms}\times \left(N_{sym}-1\right)$$
  (8) $$T_{longGI\;long\_preamble}=T_{Preamble\&Header}+40\times N_{LTF}+T_{Syml}\times N_{sym}$$

*T_Preamble&Header_* is given in Table 3 for the different configuration setups, *T_Syml_* is the duration of a symbol with the long GI and *T_Syms_* corresponds to the duration of a symbol with the short GI. *N_LTF_* corresponds to the number of long training symbols, which depends on the number of SS. Without Space-Time Block Coding (STBC), *N_LTF_* equals the number of spatial streams, except for three SS, in which case four training symbols are required. *N_sym_* is the number of symbols and is given in Equation (9):

(9) $$N_{symAH}=\lceil \frac{8+6\times N_{ES}+8\times K\times \left(L_{Header}+L_{data}\right)+\left(K-1\right)\times L_{deli}\times 8}{NDBPS}\rceil$$

$L_{deli}$ is the size of the delimiter between aggregated frames (4 Bytes). *T_BA_* calculation employs previously exposed *T_DATA_* equations but a frame of 32 Bytes is considered instead of *L_Header_* + *L_data_*. *N_ES_* and *N_DBPS_* depend on the MCS chosen and are fixed in the standard specification.

We consider data frames with maximum payload size of 1500 Bytes to build the MPDU aggregation (A-MPDU). Up to 64 individual frames are allowed to assemble an A-MPDU. Note, however, that the standard imposes other restrictions that may reduce the number of aggregated frames carried by an A-MPDU. IEEE 802.11ah presents a maximum length for an A-MPDU of 511 symbols and a maximum duration of 27.930 ms. On the other hand, IEEE 802.11n allows up to 65,535 Bytes, whereas IEEE 802.11ac is able to deal with 1,048,575 Bytes of maximum length. In both amendments, the maximum frame duration is of 5.484 ms.

**Figure 2:**
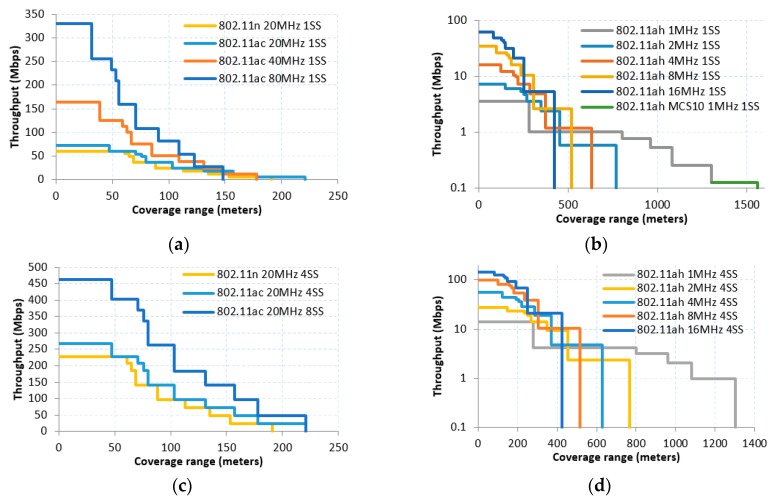
Macro deployment A-MPDU throughput vs. coverage range in IEEE 802.11: (**a**) shows the throughput using 1 SS for 802.11n and 802.11ac; (**b**) exposes the throughput for 802.11ah in 1, 2, 4, 8, 16 MHz CBW with 1 SS, highlighting the new MCS10 with 1 SS; (**c**) depicts the throughput using 4 and 8 SS for 802.11n and 802.11ac, respectively; (**d**) highlights the throughput for 802.11ah using 4 SS.

**Figure 3:**
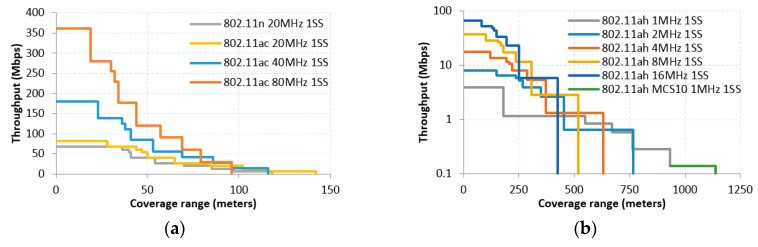

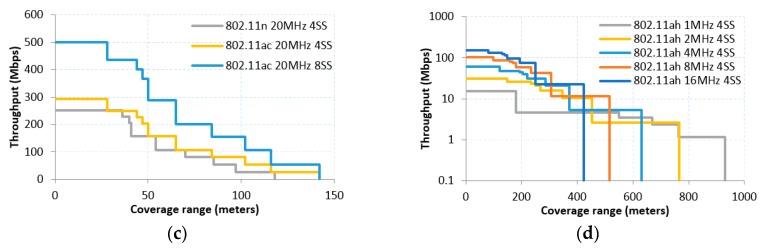
Indoor A-MPDU Throughput vs. coverage range in IEEE 802.11: (**a**) highlights the throughput using 1 SS for 802.11n and 802.11ac; (**b**) shows the throughput for 802.11ah in 1, 2, 4, 8, 16 MHz CBW with 1 SS, also exposes the throughput on 1MHz CBW and MCS10 with 1 SS; (**c**) depicts the throughput using 4 and 8 SS for 802.11n and 802.11ac, respectively; (**d**) highlights the throughput for 802.11ah using 4 SS.

Different from Hazmi et al. [13], who utilize a Bit Error Rate (BER) model for different MCS, we use the minimum receiver sensitivity established in the proposed IEEE 802.11ah amendment (cf. Tables 23–31—Receiver minimum input level sensitivity in [2]). That is, for each distance and propagation model considered, we assume the transmitter is using the fastest available MCS, the minimum sensitivity of which is larger than the received power at that distance. For that reason Figure 2 and Figure 3 show a stepped relationship between throughput and coverage range.

As expected, using the most robust MCS leads to increased coverage and more reliable communication, while employing higher order MCS, the benefit of the higher data rate in the communication scenario can be observed (cf. Figure 2 and Figure 3).

The use of sub 1GHz frequency band, together with the new and more robust modulation MCS10 provide benefit to IEEE 802.11ah in achieving the long range feature, i.e., IEEE 802.11ah amendment can operate under macro deployment scenario and can achieve a coverage range of up to 1500 m. The same PHY configuration can reach up to 900 to 1100 m in different indoor scenarios.

Hence, in terms of coverage, there is seven-fold improvement using IEEE 802.11ah with the most robust MCS with respect to best sub-6 GHz amendment result (IEEE 802.11ac, 20 MHz, with 1 SS).

Furthermore, the improvement obtained by the new MCS10 in the IEEE 802.11ah case is around 15% for distance reached in macro deployment in comparison with the lowest MCS (MCS0) with 1 SS, and around 20% in indoor case. Besides, the use of more than 1 SS improves the throughput up till 95% when employing four SS, but in turn reduces the coverage range considerably. It is also important to highlight the fact that improving range results in throughput performance decrease. However, the throughput achieved by the IEEE 802.11ah in the limit of its coverage can still reach the 100 kbps, which can be enough for most of IoT applications.

It is also worth mentioning that a higher throughput performance can be obtained for IEEE 802.11ah employing two 8 MHz or four 4 MHz channels instead of one 16 MHz channel. First, note that the use of larger CBW improves the transmission efficiency since it allows the use of a larger proportion of data subcarriers (pilot, guard subcarriers are the same regardless of the CBW used). However, the required receiver minimum input sensitivity also increases by using larger CBW, thus a better signal quality is needed at the receiver to complete a successful reception. In this way, for long distances, it results in a more profitable practice to use, for example, 16 channels of 1 MHz CBW instead of 1 channel of 16 MHz CBW; with high signal quality in reception, the larger bandwidth becomes a better option due to the better proportion of data/pilot OFDM carriers.

## 4. IoT Applications

The use of Information and Communication Technologies (ICT) as an enabler of smart cities creates the concept called Urban Automation Networks (UANs), which allows a wide spectrum of applications focused in smart cities, such as garbage collection, lighting control, green zone management, environmental control, parking availability, street traffic, utility infrastructure and security. All aforementioned applications can be included within the IoT applications framework. In addition, there are many other important applications available for IoT, such as multimedia and smart/e-health applications, smart metering, smart green and integrated transport [14], home automation, consumer services, smart grids [15], smart automotive and transit, smart logistic and supply chain, smart oil, gas manufacturing and industrial applications.

Building home automation consists on the automatic centralized control of a building in areas such as Heating, Ventilating and Air-Conditioning (HVAC), lighting, safety and security systems. Also, smart metering applications are focused on smart grids, including on demand and periodical meter reading, load management and electric service prepayments. Multimedia (audio and video devices, such as surveillance cameras or wireless speakers are not commonly considered within the IoT, but they can be used as sensors/actuators) and smart/e-health applications include phone conversations and video transmissions for emergency notification, transference of high resolution images, and smart monitoring on biometrical signals, such as electroencephalography (EEG), electrocardiography (ECG) and blood pressure (BP).

### Meeting the Requirements of IoT Applications

We present an analytical study to evaluate the viability of IEEE 802.11ah as the basis of different IoT applications by confronting the application requirements and the IEEE 802.11ah capabilities. We collect a selection of typical IoT applications, dividing them into smart applications and multimedia and smart/e-Health applications. The smart applications are further divided in two categories according to their time-related requirements: permanent connectivity and event-based applications (highlighted in Table 4). Multimedia and smart/e-Health applications (signified in Table 5) are classified by type, namely audio, video, data and biometrics. Table 4 and Table 5 show the minimum number (i.e., worst case) of stations (STAs) each IEEE 802.11ah AP can support while meeting the requirements of different IoT applications.

In all of the aforementioned IoT applications, we expose the expected number of devices that an IEEE 802.11ah standard AP can support over different distances (i.e., less than 1 km, 500 m and 250 m). In order to do that, we consider the typical data size and aggregated data rate requirements for different applications found in the literature (e.g., [15,16]). In each case, we also assume the fastest MCS (among the set of mandatory MCS) that can be reached at those distances, according to the minimum receiver sensitivity set in the IEEE 802.11ah specification (cf. Tables 23–31—Receiver minimum input level sensitivity in [2]). This explains why larger cells admit less users (larger distances require more robust and, therefore, slower modulations).

Our evaluation scenarios are conformed by multiple IEEE 802.11ah transmitters or STAs and one receiver (AP). In order to set a reliable lower bound, we assume the most demanding case; that is, all STAs are active and willing to transmit at the same time (i.e., saturation conditions). We start the evaluation with one STA and then we keep adding new STAs until the provided layer-2 throughput ceases to meet the requirements of the application, i.e., the obtained throughput per station is below the data rate required by the application. The throughput as a function of the number of contending STAs is computed according to the well-known Bianchi’s analytical model [17] and considering IEEE 802.11ah basic access parameters. Note that the specific use of IEEE 802.11ah mechanisms, such as RAW, will improve the efficiency in the radio channel access, thus allowing an increase in device density and in the number of STAs served by one AP. Also note that we are not considering any multiplexing gain when, for most applications, it is unlikely that all associated STAs are active simultaneously. As a rule of thumb, the total number of associated devices supported could be obtained by dividing the number of devices reported in Table 4 and Table 5 by the expected duty cycle of the application, measured during the hours of maximal activity. In many applications where the duty cycle is very small (e.g., few transmissions per hour or per day), the limit in the number of supported devices is actually determined by the AID field (i.e., near 8200 devices per AP) and not by the achieved throughput. For the sake of example, let us assume that the distribution automation application requires each connected device to transmit 600 Bytes (4 frames with a payload of 150 Bytes each, cf. Table 4) every 5 s. The resulting duty cycle considering the slowest bit rate (i.e., 150 kbps at MCS10) is <1.3%. According to Table 4, the maximum number of simultaneous transmitters at the largest distance is 55 and, therefore, we could admit up to 4200 associated devices; however, note that with 4200 transmitters, the probability of having 56 or more simultaneous contenders (i.e., probability of having congestion) is relatively high, at 30%. Therefore, in order to reduce congestion, we suggest that the number of admitted stations is reduced to 80% or less (e.g., 3300); in such case, the probability of congestion is reduced to less than 2% (assuming that stations behave as independent ON-OFF state machines, the number of simultaneous transmitters and congestion probability can be obtained by treating the system as an *M/M/C*, where *C* corresponds to the number of supported devices reported in Table 4 and Table 5).

It is also apparent, how in circumscribed cases (backhaul, firmware, EHR, video and image applications), the use of frame aggregation is a key enabler, necessary to meet throughput requirements.

Finally, we would like to highlight the fact that most of the technologies presented in Table 1, do not meet throughput requirements of most of the IoT applications considered in this Section 4 when providing enough coverage and supported users, or fail to provide a decent coverage when meeting throughput requirements. Note that, in comparison with other notable technologies contenders for IoT, which offer data rates from 1 kbps up to 1 Mbps, IEEE 802.11ah presents a wider range of available operating rates that go from 150 kbps up to 346 Mbps.

A clear example is provided with multimedia applications. The multimedia term has not been usually associated with the IoT paradigm due to the lack of capacity of traditional IoT solutions for supporting the required bit rates. With the exposed analysis we show that IEEE 802.11ah enables the IoT to adopt new use cases involving the transmission of multimedia data (i.e., audio/video), thus making the link between multimedia and IoT applications now possible.

## 5. Application and Infrastructure Costs

In order to provide a more complete view of the viability of an IEEE 802.11ah-based IoT infrastructure, in this section we give an approximation of its costs. We assume a highly dense scenario of 1 km^2^ populated by 10,000 IoT devices, i.e., sensors/actuators connected together in the same area. We calculate the total infrastructural cost to cover 6 and 12 years of operation (short and medium term-operation). We focus this analysis on the costs of the radio interfaces, disregarding the costs of the site (placement and installation of the APs) and the cost of the device, which will be comparable regardless of the wireless technology chosen.

A typical scenario based on legacy IEEE 802.11 technology, would require, at least, 50 APs: first, we assume enterprise-level APs supporting up to 200 connected devices per AP and an effective coverage radius of 80 m to serve the whole 1 km^2^ area. Second, we consider 20 USD per radio interface and 500 USD per AP. The investment on the aforementioned assets falls under the denominated CAPital EXpenditure (CAPEX, the investment needed to acquire the elements conforming the infrastructure on a project). The OPeration EXpenditure (OPEX, the investment that will be needed to maintain the installations in working conditions) can be estimated as the 10% of the CAPEX plus the salaries of the IT staff who will operate and manage the network. Noting that the OPEX is calculated per year, the project generates a total outlay of 740,000 USD in a six year project and an investment of 1,200,000 USD in a twelve year project.

On the other hand, we analyze the same scenario based on IEEE 802.11ah technology with the caveat that there are still no IEEE 802.11ah products available in the market and, therefore, precise price ranges cannot be given. We assume the same requirements presented previously. In terms of coverage, just two IEEE 802.11ah APs would be enough. However, in order to guarantee a good service to 10,000 IoT devices, four APs are recommended, each of which can cover a radius of less than 300 m (IEEE 802.11ah APs can reach more than 1km in typical outdoor deployments) and can serve 2500 devices (the maximum number of devices allowed in a IEEE 802.11ah AP is ~8000). As explained, the sensor/actuator hardware will cost the same amount as in the previous case. However, IEEE 802.11ah NICs are expected to be cheaper since they are intended to be integrated in low-cost small devices (assume 15 USD per radio interface); on the other hand, APs are more expensive (assume 1,000 USD per AP). With the same criteria to assess the OPEX, the total cost for a 6 year project with IEEE 802.11ah would be of 540,000 USD and of 940,000 USD in a twelve year project (close to 25% cheaper).

In the same scenario, we estimate deployment costs of other IoT communication alternatives, such as the proprietary solutions LoRaWAN or SigFox. In this case, a sensor radio costs around 10 USD. Three base stations are going to be needed to support 10,000 devices, with an approximately price of 6000 USD each one. Thus, following the same rules for OPEX computation, the total cost would be around 484,000 USD for a six year project and around 850,000 USD for a twelve year project. Those alternatives offer lowest implementation costs in comparison to IEEE 802.11ah technology, but the higher complexity of LoRaWAN/SigFox interconnection and the limited available bandwidth are the limitations holding back a wider adoption in these IoT technologies.

In addition, with regard to the IoT scenario based on cellular technologies, each sensor radio that is going to be connected to the operator infrastructure has an approximate cost of 50 USD. In this case, for the OPEX computation, the 20% of the CAPEX is usually considered, due to the addition of data plane maintenance costs. Thus, the estimated OPEX would be around 1,100,000 USD for a 6 year project and around 2,300,000 USD for a 12 year project, thus making cellular technology the most expensive approach.

## 6. Conclusions

The potential coverage at reasonably high rates exhibited by IEEE 802.11ah makes it an attractive alternative in fulfilling the needs of future IoT communications. In this article, we provide a comparison between different technologies contending to cover the IoT communications framework, and thus indicate IEEE 802.11 technology as one of the strongest contenders.

We evaluate the main characteristics and benefits provided in terms of throughput and transmission range by the most notable IEEE 802.11 specifications compared to IEEE 802.11ah amendment. The analysis of the results presents IEEE 802.11ah with more than 8 times improvement in coverage range against any other IEEE 802.11-based amendment and shows that it can provide throughput close to 100kbps in the worst case, which is enough to cover most IoT applications.

We give a thorough analysis of the requirements of many typical IoT applications (classified as permanent connectivity, event-based applications, audio, video, data and biometrics), assessing the number of supported devices per AP, with up to 1 km of coverage. In the cases where the required coverage distance is larger than 1km, IEEE 802.11ah can be used to build a multi-hop distribution system.

We also provide an analysis of the implementation and infrastructure costs that make IEEE 802.11ah very attractive in front of other IEEE 802.11 specifications and competing wireless technologies. Overall, the expected performance of IEEE 802.11ah asks for a remarkable place in the IoT landscape.

## Figures and Tables

**Table 1:** Notable technologies contenders for IoT.

Feature	IEEE 802.11 (n/ac)	IEEE 802.11ah	ZigBee/802.15.4e	BLE	3GPP MTC	LPWAN	
						LoRaWAN	SigFox
Frequency band (GHz)	Unlicensed 2.4, 5 GHz	Unlicensed 900 MHz	Unlicensed 868/915 MHz 2.4 GHz	Unlicensed 2.4 GHz	Licensed <5 GHz	Unlicensed 867–928 MHz	Unlicensed 868–902 MHz
Data Rate	6.5–6933 Mbps	150 kbps–346 Mbps	<250 kbps	<1 Mbps	<1 Mbps	<25 kbps	<1 kbps
Coverage range	<200 m	<1.5 km	<100 m	<50 m	<100 km	<20 km	<40 km
Power consumption	Medium	Low	Low	Low	Low	Low	Low
Number of devices supported	2007	8000	65,000	Unlimited *	>100,000	>100,000	>1,000,000

* BLE supports an unlimited number of devices, this depends on the configured address space.

**Table 2:** Key MAC features within each amendment.

Notable Features		802.11-2007	802.11n	802.11ac	802.11ah
Backwards compatibility		X	X	X	
DCF		X			
PCF		X			
HCF	HCCA	X	X		X
	EDCA	X	X	X	X
TXOP	Forward	X	X	X	X
	RD protocol		X	X	X
	BDT				X
RID					X
Frame Aggregation			X	X	X
Block ACK		X	X	X	X
Multi User (MU) Aggregation				X	X
Null Data Packet (NDP)			X	X	X
Group-ID				X	X
BSS color					X
Dynamic Bandwidth Management				X	
Subchannel Selective Transmission					X
Traffic Indication Map (TIM)		X	X	X	X
Delivery Traffic Indication Map (DTIM)			X	X	X
Target Wakeup Time					X
Grouping of Stations					X
Hierarchical AID					X
Dynamic AID reassignment					X
Restricted Access Window (RAW)					X
Group sectorization					X
Relay operations					X
Power saving at AP					X
Low power mode of operations					X

**Table 3:** MAC/PHY Parameters.

Specification	SIFS (µs)	DIFS (µs)	T_Preamble &Header_ (µs)	MAC&LLC Header Size (Bytes)	Signal Extension (µs)	T_Sym_ (µs)	T_Slot_ (µs)	CW_min_	CW_max_
802.11ah CBW 1 MHz	160	264	560	26 (Short) 36 (Long)	n/a	40 (long GI) 36 (short GI)	52	15	1023
802.11ah Short Preamble CBW 2, 4, 8 and 16 MHz	160	264	240	26 (Short) 36 (Long)	n/a	40 (long GI) 36 (short GI)	52	15	1023
802.11ah Long Preamble CBW 2, 4, 8 and 16 MHz	160	264	320	26 (Short) 36 (Long)	n/a	40 (long GI) 36 (short GI)	52	15	1023
802.11ac	16	34	40	36	n/a	4	9	15	1023
802.11n 2.4 GHz	10	28	36	36	6	4	9	15	1023
802.11n 5 GHz	16	34	36	36	0	4	9	15	1023

**Table 4:** Number of supported STAs per IEEE 802.11ah AP for different smart applications.

	Application	Description	Average Payload Size (Bytes)	Average Aggregate Data Rate (Kbps)	Supported Devices at <1 km (Outdoor)	Supported Devices at <500 m (Outdoor)	Supported Devices at <250 m (Indoor)
Permanent connectivity applications	Home/Building automation	Sensitive delay applications, including services to manage different commodity infrastructure, remote control of industrial facilities, smart cities applications, etc.	100	15–30	1250	2100	2500
	On-demand meter reading		100	40–180	250	1000	1200
	Distribution Automation		150	60–480	55	300	400
	Electric service prepayment		50–150	30–90	725	2000	2100
	Service on/off switch		25	5–10	1600	2400	2600
	Security (sensors, alarms).		100	40–180	250	1050	1150
	Backhaul/core/metro networks *		1500	240–4100	1	6	17
	Parking Availability		100	40–180	250	1050	1150
	Street traffic		100	40–180	250	1050	1150
Event-based applications	Multi-interval meter reading	Delay-tolerant where data is collected infrequently (multiple times per day) applications, including all non-critical applications not requiring permanent connectivity such as scheduled reporting of bulk measurements.	100	<1	4200	5000	5300
	Firmware Updates +		1500	45–250	400	1800	2500
	Garbage Collection		100	<1	4200	5000	5300
	Lighting Control		100	<1	4200	5000	5300
	Green zone management		100	<1	4200	5000	5300
	Environmental Control		64	<1	4200	5000	5300
	Utility infrastructure		100	<1	4200	5000	5300

* The numbers of the backhaul application are provided assuming frame aggregation, with which IEEE 802.11ah is capable of meeting the minimum throughput requirements of the backhaul application. Note that wireless backhaul application consists in a network of point-to-point links, where the required number of supported STAs per link is 1 (plus the AP). A number of STAs X > 1 means that X/2 bidirectional links can coexist in the same channel and still meet the throughput requirements. Also note that, in this particular application, we can safely assume MxM MIMO capable nodes, which have the potential to multiply by M the throughput obtained (M ≤ 4). + The firmware application also needs the use of the frame aggregation feature to allow higher throughput for timely bulk data transfer of, typically, 400-2000KBytes.

**Table 5:** Number of supported STAs per IEEE 802.11ah AP for different multimedia and smart/e-health applications.

	Application	Description	Average Payload Size (Bytes)	Average Aggregate Data Rate (kbps)	Supported Devices at <1 km	Supported Devices at <500 m	Supported Devices at <250 m
Audio	Audio 1 Codec G723.1 Rate 6.4 kbps	In these applications, a variety of codecs are available depending on the audio quality required.	100	80–600	5	15	30
	Audio 2 Codec AMRx Rate 12.2 kbps		120	70–650	5	20	35
Video	Video 1 Codec H.264 Rate 500 kbps	In these applications different codecs are needed depending on the quality of the video required.	1500	500–4000	1	3	7
	Video 2 * Codec H.264 Rate 8 Mbits/s		1500	8000–25,000	-	1	3
Data	Electronic Health Record (EHR) +	Applications involving the transmission of large files in the context of smart/e-health.	1000	1000–10,000	1	5	10
	IMG 1 Low resolution lossless compression, 1024 × 768 px 24 bits/px		1500	450–2000	3	9	12
	IMG 2 ** High resolution lossless compression, 4096 × 4096 px 24 bits/px		1500	3500–20,000	1	2	6
Biometrics	Electroencephalography EEG	Applications where data is collected from the electrical signals in the human body to get representative information in the evolution of vital signs.	100	100–400	1	2	3
	Electrocardiography ECG		50	50–300	1	5	10
	Blood pressure(BP)/Pulse Oximeter (SpO${}_{2}$)		400	80–1100	25	140	320

+ Bulk data transfer applications will benefit from the use of frame aggregation. For example, with frame aggregation, IEEE 802.11ah could support up to 10 simultaneous EHR users at 600 m whereas, without aggregation, the available throughput only leaves room for one user meeting the required quality. * and ** Video 2 and IMG 2 applications will also benefit from the use of frame aggregation and of more than 1 SS; however IEEE 802.11ah is able to transmit typical quality images and video files needed for most applications.
